# Statistical analyses on inverted perovskite solar cells employing non-fullerene-based small molecule as a cathode interfacial layer

**DOI:** 10.1016/j.dib.2018.06.043

**Published:** 2018-06-22

**Authors:** Yong-Jin Noh, Ji-Ho Jeong, Seok-In Na

**Affiliations:** Professional Graduate School of Flexible and Printable Electronics and Polymer Materials Fusion Research Center, Chonbuk National University, Jeonju-si, Jeollabuk-do 561-756, Republic of Korea

## Abstract

*In this data article,* we present the influences of the solvent, concentration, and spin rates of 3,9-bis(2-methylene-(3-(1,1-dicyanomethylene)-indanone))-5,5,11,11-tetrakis(4-hexylphenyl)-dithieno[2,3-d:2’,3’-d’]-s-indaceno[1,2-b:5,6-b’]dithiophene) (ITIC) material on the performances of perovskite solar cells (PSCs). The device parameters such as open-circuit voltage (Voc), short circuit current (Jsc), fill factor (FF), and power conversion efficiency (PCE) were measured with Keithley 2400 source meter unit under 100 mW/cm^2^ (AM 1.5 G). The data in this article describe the optimization of ITIC-based PSCs and are directly related to our research article “Non-fullerene-based small molecules as an efficient n-type electron transporting layers in inverted organic-inorganic halide perovskite solar cells” (Noh et al., Submitted for publication) [1].

**Specifications Table**

**Table:** 

Subject area	*Electrical Engineering*
More specific subject area	*Perovskite Solar Cells*
Type of data	*Figure*
How data was acquired	*Keithley 2400 source meter unit under 100 mW/cm*^*2*^*(AM 1.5 G)*
Data format	*Analyzed*
Experimental factors	*Average solar cell parameters plotted using ~ 20 devices*
Experimental features	*The error bars of PSC parameters were obtained by the current density-voltage characteristics.*
Data source location	*Chonbuk National University, Jeonju-si, Jeollabuk-do, 561–756, Republic of Korea*
Data accessibility	*The data are with this article.*

**Value of the data**

• The data article presents the influence of the solvent, concentration, and spin-rate of ITIC on the PSC parameters such as Voc, Jsc, FF, and PCE.
• The data article can be used to fabricate PSCs with the ITIC-based ETLs.
• These data will be helpful to understand the fabrication of PSC with the small molecule materials as the electron transporting layer.

## 1 Data

**Fig. 1:**
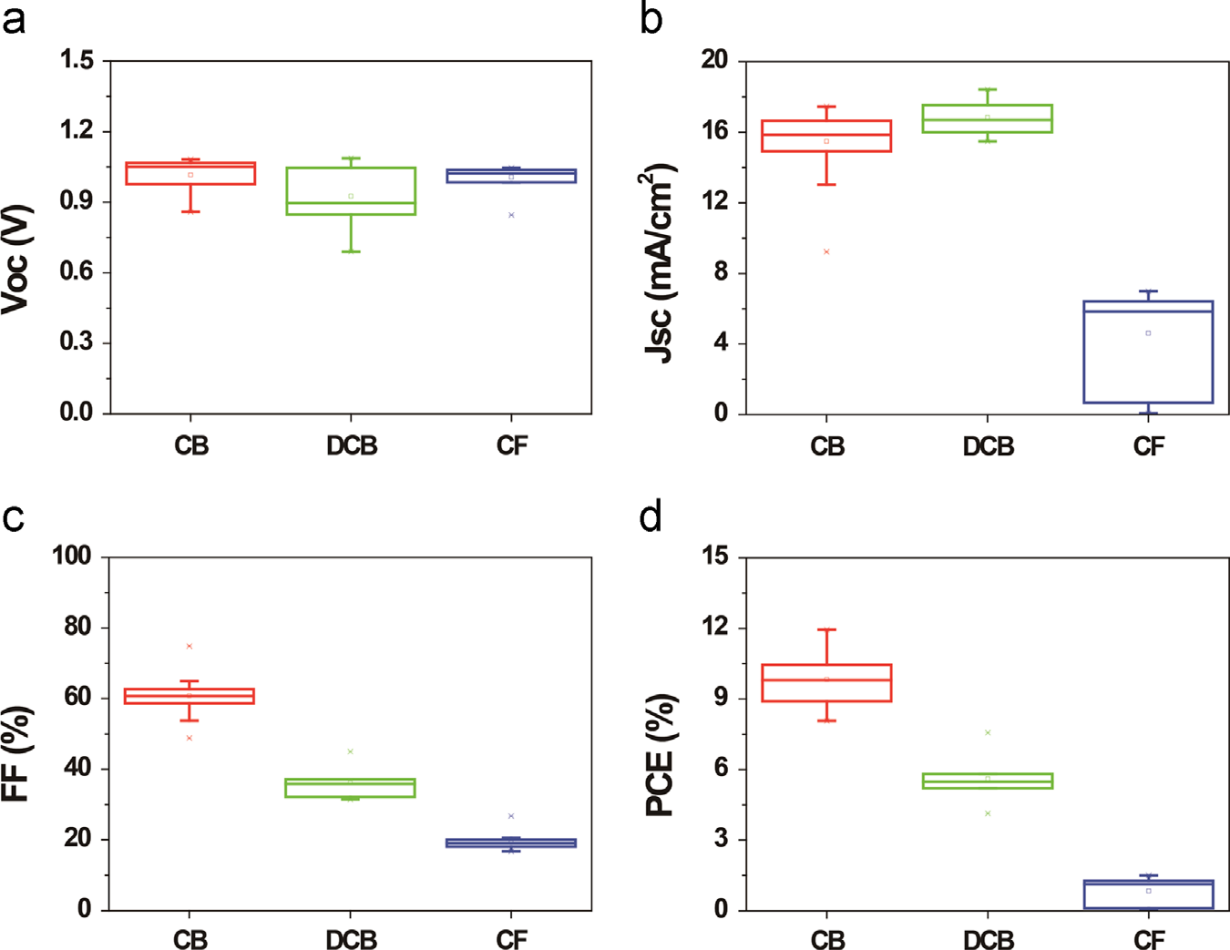
The influence of the ITIC solvent on the PSC parameters such as (a) Voc, (b) Jsc, (c) FF, and (d) PCE.

**Fig. 2:**
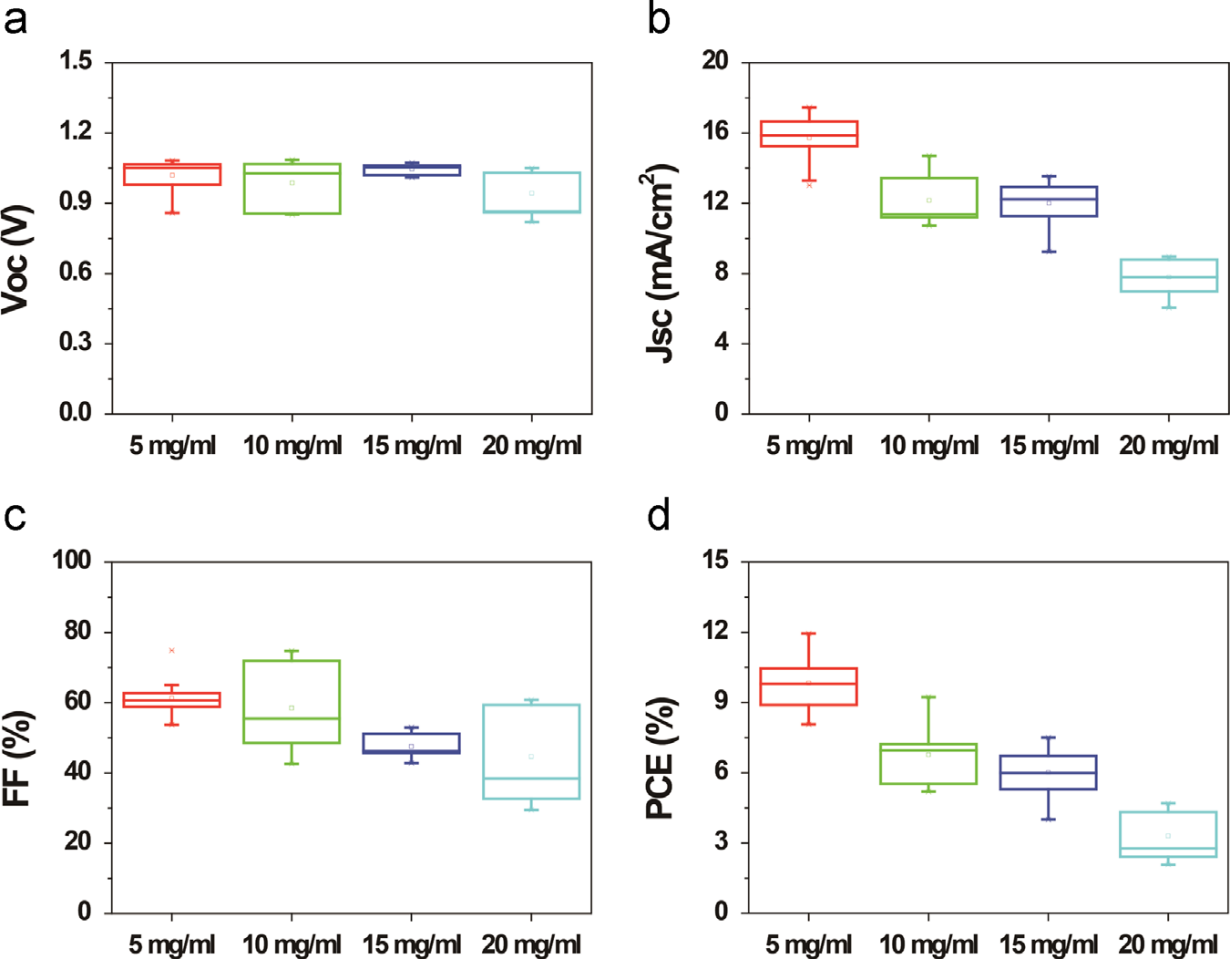
Changes in device parameters such as (a) Voc, (b) Jsc, (c) FF, and (d) PCE with increasing ITIC concentrations.

**Fig. 3:**
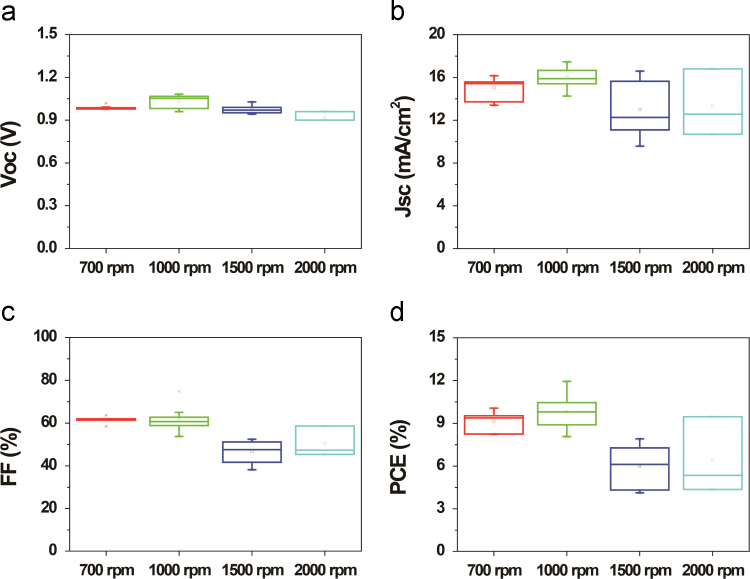
Changes in device parameters such as (a) Voc, (b) Jsc, (c) FF, and (d) PCE with increasing ITIC spin rates.

We investigated the influences of the solvents, concentrations, and spin rates of ITIC material on the PSC-parameters such as Voc, Jsc, FF, and PCE. For comparative study, we chose the three different solvents such as chlorobenzene(CB), di-chlorobenzene (DCB), and chloroform (CF), as shown in Fig. 1. The device with ITIC dissolved in CB exhibited the best PSC performance compared with the PSC with ITIC dissolved in DCB and CF. Fig. 2 showed the average parameters of PSCs with ITIC films prepared with concentration of 5, 10, 15, 20 mg ml^-1^. The PSC with 10 mg ml^-1^-based ITIC showed best efficiency. Finally, the PSCs with 10 mg ml^-1^ ITIC spin-coated at 700, 1000, 1500, and 2000 rpm fabricated, as shown in Fig. 3. The PSC with 1000 rpm-coated ITIC showed the maximum device performance.

## 2 Experimental design, materials and methods

The fabrication of PSCs was performed using the method described in our previous paper [1]. The photovoltaic characteristics of PSCs prepared with the various solvent, concentrations, and spin-rate were measured using a Keithley 2400 instrument under 100 mW/cm^2^ solar illumination generated by a 450 W Oriel solar simulator at AM (air mass) 1.5 G condition.
