# Supplementary material for: Statistical analyses on inverted perovskite solar cells employing non-fullerene-based small molecule as a cathode interfacial layer
Source: Data Brief. 2018 Jun 22;19:1643–6. doi: 10.1016/j.dib.2018.06.043 (PMC6141261; doi:10.1016/j.dib.2018.06.043)
Supplement: Supplementary file 1 — Supplementary material [file mmc1.pdf]

Professional Graduate School of Flexible and Printable Electronics,
Department of Flexible and Printable Electronics,
Chonbuk National University,
664-14, Deokjin-dong, Jeonju-si, Jeollabuk-do, 561-756,
Republic of Korea ,

nsi12@jbnu.ac.kr
Tel : +82-63-270-4465
Fax : +82-63-270-2341

July 02, 2018

Dear Editor:

We accepted the manuscript entitled, "Statistical analyses on inverted perovskite solar cells employing non-fullerene-based small molecule as a cathode interfacial layer" written by Yong-Jin Noh, Ji-Ho Jeong, and Seok-In Na in ***Data in Brief.***

The authors complied the Elsevier's ethics in publishing; submission of an article implies that the work described has not been published previously (except in the form of an abstract or as part of a published lecture or academic thesis), that it is not under consideration for publication elsewhere, that its publication is approved by all authors and tacitly or explicitly by the responsible authorities where the work was carried out. Submission also implies that, if accepted, it will not be published elsewhere in the same form, in English or in any other language, without the written consent of the Publisher. In addition, the named authors of this manuscript have NO conflict of interest, financial, or otherwise.

Author name	Author signature	Date
Yong-Jin Noh		July 02, 2018

Author name	Author signature	Date
Ji-Ho Jeong		July 02, 2018

Author name	Author signature	Date
Seok-In Na		July 02, 2018

Sincerely yours,

Prof. Seok-In Na

Professional Graduate School of Flexible and Printable Electronics,

Department of Flexible and Printable Electronics,

Chonbuk National University, 664-14, Deokjin-dong, Jeonju-si, Jeollabuk-do, 561-756,

Republic of Korea

TEL : +82-63-270-2335, Fax : +82-63-270-2341, C.P : +82-10-3070-2895

E-mail : nsi12@jbnu.ac.kr
